# Reactions of Medicinal Gold(III) Compounds With Proteins and Peptides Explored by Electrospray Ionization Mass Spectrometry and Complementary Biophysical Methods

**DOI:** 10.3389/fchem.2020.581648

**Published:** 2020-10-21

**Authors:** Lara Massai, Carlotta Zoppi, Damiano Cirri, Alessandro Pratesi, Luigi Messori

**Affiliations:** ^1^Department of Chemistry, University of Florence, Florence, Italy; ^2^Department of Chemistry and Industrial Chemistry, University of Pisa, Pisa, Italy

**Keywords:** anticancer metal complexes, gold, protein interaction, mass spectrometry, cytotoxic compounds

## Abstract

Electrospray ionization mass spectrometry (ESI MS) is a powerful investigative tool to analyze the reactions of metallodrugs with proteins and peptides and characterize the resulting adducts. Here, we have applied this type of approach to four experimental anticancer gold(III) compounds for which extensive biological and mechanistic data had previously been gathered, namely, Auoxo6, Au_2_phen, AuL12, and Aubipyc. These gold(III) compounds were reacted with two representative proteins, i.e., human serum albumin (HSA) and human carbonic anhydrase I (hCA I), and with the C-terminal dodecapeptide of thioredoxin reductase. ESI MS analysis allowed us to elucidate the nature of the resulting metal–protein adducts from which the main features of the occurring metallodrug–protein reactions can be inferred. In selected cases, MS data were integrated and supported by independent ^1^HNMR and UV–Vis absorption measurements to gain an overall description of the occurring processes. From data analysis, it emerges that most of the investigated gold(III) complexes, endowed with an appreciable oxidizing character, undergo quite facile reduction to gold(I); the resulting gold(I) species tightly associate with the above proteins/peptides with a remarkable selectivity for free cysteine residues. In contrast, in the case of the less-oxidizing Aubipyc complex, the gold(III) oxidation state is conserved, and a gold(III) fragment still containing the original ligand is found to be associated with the target proteins. It is notable that the C-terminal dodecapeptide of thioredoxin reductase containing the characteristic –Gly–Cys–Sec–Gly metal-binding motif is able in all cases to trigger gold(III)-to-gold(I) reduction. Our investigation allowed us to identify in detail the nature of the gold fragments that ultimately bind the protein targets and determine the exact binding stoichiometry; some insight on the reaction kinetics was also gained. Notably, a few clear correlations could be established between the structure of the metal complexes and the nature of the resulting protein adducts. The mechanistic implications of these findings are analyzed and thoroughly discussed. Overall, the present results set the stage to better understand the real target biomolecules of these gold compounds and elucidate at the atomic level their interaction modes with proteins and peptides.

## Introduction

During the last two decades, a number of studies highlighted the importance of gold compounds as a new family of cytotoxic agents with the potential of becoming new effective anticancer drug candidates endowed with original mechanisms of action and a peculiar spectrum of antitumor activities (Nobili et al., 2010; Zou et al., 2015). Indeed, a variety of gold(III) and gold(I) compounds, bearing different structural motifs, were reported to cause extensive cell death *in vitro* in numerous cancer cell lines, with a remarkable selectivity for cancer over normal cells (Casini et al., 2009). From the mechanistic studies conducted so far, the modes of action and the targets of antiproliferative gold compounds appear to be multifaceted and deeply distinct from those of clinically established platinum compounds (i.e., cisplatin); yet the “true” molecular mechanisms of medicinal gold compounds remain largely unexplored (Fiskus et al., 2014). A growing body of evidence suggests that a few selected protein targets such as the enzyme thioredoxin reductase (Scalcon et al., 2018) and some transcription factors with zinc finger motifs (Abbehausen, 2019) mediate primarily the biological effects of cytotoxic gold compounds. This prompted investigators to analyze in detail the reactions of medicinal gold compounds with proteins. A systematic investigation on the interactions of the reference gold(I) drug auranofin with a series of model proteins, i.e., human serum albumin, carbonic anhydrase, superoxide dismutase, and hemoglobin, was recently carried out in our laboratory through mass spectrometry, and the resulting adducts could be characterized in molecular detail (Pratesi et al., 2016, 2018, 2019; Magherini et al., 2018; Cirri et al., 2019; Zoppi et al., 2020). These studies revealed that auranofin targets quite selectively the free cysteine residues of proteins (Pratesi et al., 2018; Zoppi et al., 2020).

Here, we have extended this kind of investigation to four representative gold(III) compounds for which extensive biological and mechanistic data have been gathered in the last years, namely, Auoxo6, Au_2_phen, AuL12, and Aubipyc (Marzano et al., 2011; Gabbiani et al., 2012a,b). More precisely, the remarkable antiproliferative effects of the structurally related Auoxo6 and Au_2_phen complexes were highlighted since the late 2000s by our research group in connection with the group of Cinellu et al. (2010), Casini et al. (2006). The complex AuL12 was developed and characterized, in Padua, by the research group of Marzano et al. (2011); Aubipyc was prepared in Sassari by the research team of Marcon et al. (2002), and its biological profile was later analyzed in connection with our research group (Marzo et al., 2015; Massai et al., 2016a). Here, we have studied the reactions of the above-mentioned gold(III) compounds with two representative proteins, i.e., HSA and hCA I; in addition, we have decided to perform the same interaction studies against the C-terminal redox-active domain of thioredoxin reductase, an important enzyme involved in cellular redox homeostasis regulation that is the putative target for gold compounds. Our investigation mainly relies on MS determinations, but MS results are independently supported by other biophysical techniques such as ^1^HNMR and UV–Vis. Notably, the results of this multi-technique approach turned out to be nicely consistent each other and allowed us to identify for each of the four selected gold complexes the precise nature of the metallic fragments that bind the investigated biomolecules. Characteristic trends in the analyzed metallodrug/biomolecule reaction could be identified and correlated to the structural and chemical features of the study compounds.

## The Panel of Gold(III) Complexes and the Investigative Strategy

**Figure 1:**
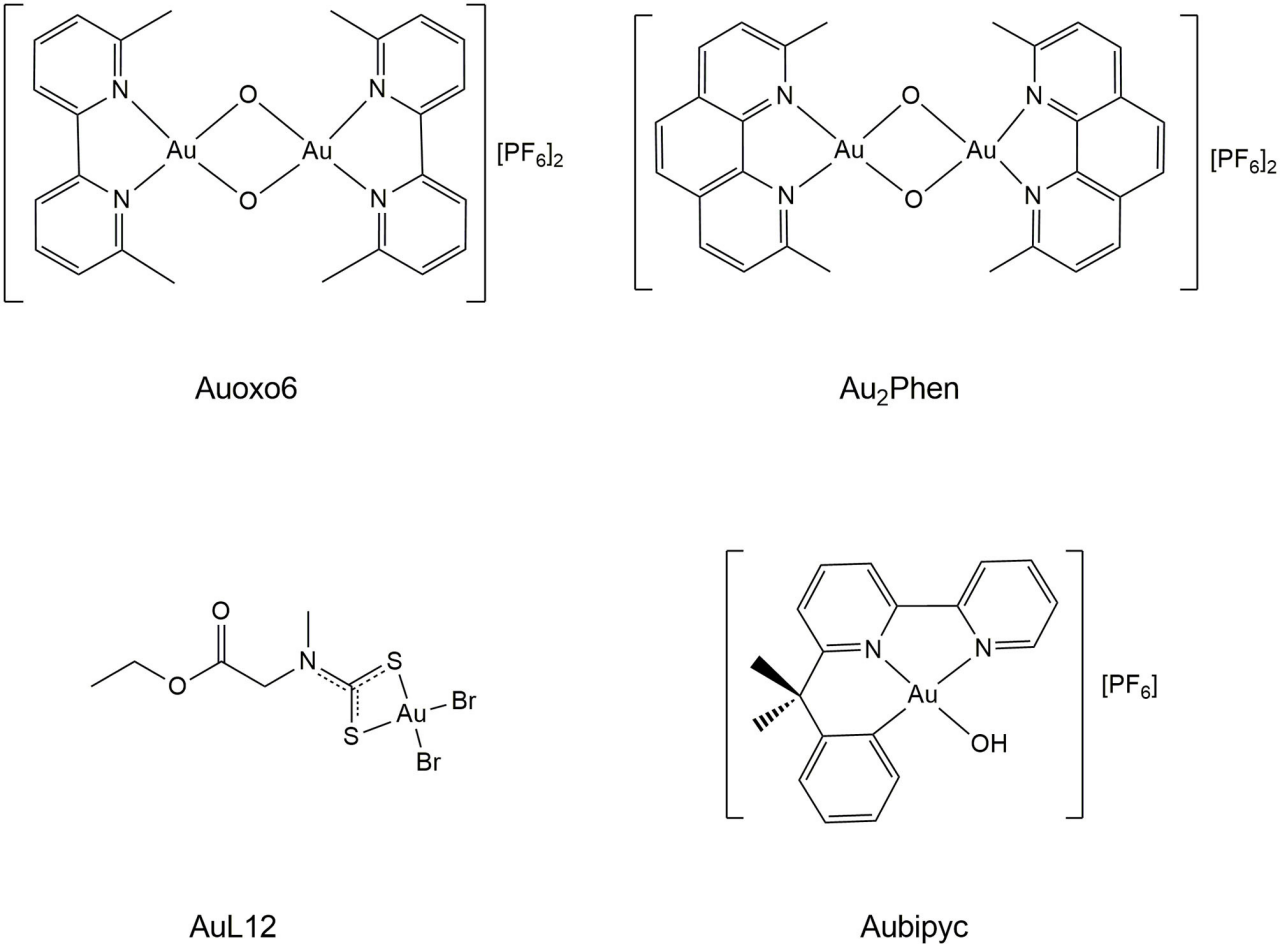
The panel of the studied gold(III) compounds.

The chemical formulas of the study compounds are synoptically shown in Figure 1.

Auoxo6 and its analogous Au_2_phen, with two 2,9-dimethyl phenanthrolines in the place of two bipyridines, are binuclear Au(III) coordination compounds with a dioxo-bridge, which links the two gold(III) centers. Their main feature is the presence of an extended, roughly planar system containing the Au_2_O_2_ diamond core and the two aromatic moieties. Crystal structures for these compounds were previously determined (Gabbiani et al., 2008; Cinellu et al., 2010). These two gold(III) compounds are strictly related to each other from the structural point of view but greatly differ in their aqueous solubility, with Auoxo6 being far more soluble than Au_2_Phen. Notably, both these gold(III) compounds manifest a frank oxidizing character and tend to undergo facile reduction to gold(I) or, alternatively, to elemental gold. Their reactions with biologically reducing agents such as glutathione and ascorbate were earlier investigated, and quick gold(III) reduction was indeed documented (Gabbiani et al., 2012b). However, in the absence of reducing agents, these gold(III) complexes are stable under physiological conditions for several hours or even days and are thus well suitable for pharmaceutical application (Gabbiani et al., 2012b).

AuL12 is a mononuclear gold(III) complex developed in the group of Prof. Dolores Fregona, in Padua, that revealed very promising anticancer properties both *in vitro* and *in vivo* (Ronconi et al., 2012; Nardon and Fregona, 2015). AuL12 consists of a square-planar gold(III) center with a bidentate dithiocarbamate ligand and two bromide ligands. Notably, AuL12 was shown to behave as a prodrug upon releasing its bromide ligands (Pratesi et al., 2019). Significant oxidizing properties were also documented for this gold(III) complex (Nardon et al., 2017).

Aubipyc is a gold(III) cyclometalated derivative of 6-(1,1-dimethylbenzyl)-2,2′-bipyridine, where the tetracoordinated square planar gold(III) center is surrounded by the N,N,C sequence of donor atoms from the terdentate bipyridine ligand plus an oxygen atom from the hydroxo ligand (Cinellu et al., 1996). At variance with the above gold(III) complexes, the direct carbon-to-metal bond greatly stabilizes the gold(III) center in Aubipyc; a notable consequence is that Aubipyc is not reduced by glutathione (Gamberi et al., 2014).

Some years ago, the *in vitro* anticancer properties of these four gold(III) complexes were evaluated comparatively in a standard 36-cancer cell line panel available in Oncotest (Freiburg, Germany) (Casini et al., 2009). The COMPARE algorithm applied to the analysis of the obtained growth inhibition data revealed that the profiles of Au_2_phen are very similar to those of Auoxo6, in agreement with their pronounced structural analogy (Cinellu et al., 2010). Tentatively, through bioinformatic analysis, the cytotoxicity patterns obtained for Au_2_phen and Auoxo6 were mainly ascribed to inhibition of histone deacetylase. At variance, Aubipyc was found to be only moderately effective in the Oncotest panel (Casini et al., 2009). Also, AuL12 was confirmed to be highly cytotoxic in the Oncotest panel, with a mode of action resembling antiproteasomal agents (Casini et al., 2009; Zhang et al., 2010). Interestingly, the correlations in the antiproliferative profiles between these gold compounds and cisplatin were very poor, implying again the occurrence of profoundly different modes of action. On the whole, COMPARE analysis of these gold compounds suggested that a variety of proteins might be reliable biomolecular targets and thus account for the observed biological effects; this means that the biological actions of these gold compounds are best interpreted in terms of metalation and inactivation of a few crucial proteins that are effective cancer targets (Cinellu et al., 2010).

The biological studies were later supported by a number of proteomic studies highlighting the alterations in protein expression induced by the above gold(III) complexes (Magherini et al., 2010; Guidi et al., 2012; Gamberi et al., 2014).

During the last decade, the interactions of some of these gold(III) compounds with model proteins were also investigated from the crystallographic point of view, mostly in the research group of Prof. Antonello Merlino in Naples. Those crystallographic studies clearly supported the occurrence of gold(III)-to-gold(I) reduction in the cases of Auoxo6, Au_2_phen, and AuL12 reacting with proteins (Russo Krauss et al., 2014; Merlino et al., 2017; Ferraro et al., 2019; Giorgio and Merlino, 2020).

In the last few years, ESI MS has emerged as a powerful tool to monitor the interactions of metallodrugs with proteins at the molecular level (Merlino et al., 2017). These recent developments are described in a couple of review articles (Merlino et al., 2017; Messori and Merlino, 2017). Accordingly, we have exploited the ESI MS method to characterize in a systematic way and comparatively the interaction of the four study metallodrugs with two exemplary proteins and with the C-terminal dodecapeptide of thioredoxin reductase.

Briefly, the adopted experimental strategy was as follows. All four gold complexes were challenged with HSA, hCA I, and the TrxR dodecapeptide according to a well-established experimental protocol (Tamasi et al., 2014; Marzo et al., 2017, 2019; Michelucci et al., 2017; Pratesi et al., 2018; Ferraro et al., 2020; Zoppi et al., 2020). Different times and stoichiometries were explored to identify the best conditions to obtain adduct formation and to improve the quality of the ESI MS spectra. In addition, particular attention was paid to the kinetic aspects of these reactions.

Indeed, MS turned out to be a powerful tool to characterize the metallodrug/protein adduct that formed in the course of the above reaction. Systematic MS measurements were able to define the general patterns of reactivity between gold compounds and the model proteins; however, in selected cases, UV–Vis and NMR spectroscopies were valuable ancillary techniques to better understand specific features of the investigated systems and of the resulting adducts.

## The Electrospray Ionization Mass Spectrometry Measurements

The ESI MS measurements were performed according to the procedure developed in our laboratory, which is described in detail in the Materials and Methods (Tamasi et al., 2014; Massai et al., 2016b; Pratesi et al., 2016, 2018; Zoppi et al., 2020). Briefly, each protein sample, dissolved in ammonium acetate solution 2 × 10^−3^ M at pH 6.8, was incubated with each selected gold(III) complex for 2 or 24 h in a well-defined molar ratio; then the samples were analyzed by high-resolution ESI MS through direct infusion in the mass spectrometer upon addition of a small aliquot of formic acid. The obtained MS results are shown below for each gold complex/biomolecule pair.

Remarkably, all the selected biomolecules contain a free exposed cysteine or selenocysteine (in the case of the TrxR dodecapeptide) residue, as gold–sulfur/selenium interactions are strongly preferred according to the Pearson concept (Dabrowiak, 2012).

### The Reactions of Gold Compounds With Human Serum Albumin

HSA is characterized by the presence of 17 disulfide bonds and only one free cysteine residue (Cys34) (Rombouts et al., 2015). Interestingly, the only free cysteine (Cys34) of HSA is solvent accessible and is considered the primary binding site for gold compounds owing to the high affinity of gold to sulfur-containing ligands (Pratesi et al., 2018, 2019; Zoppi et al., 2020).

**Figure 2:**
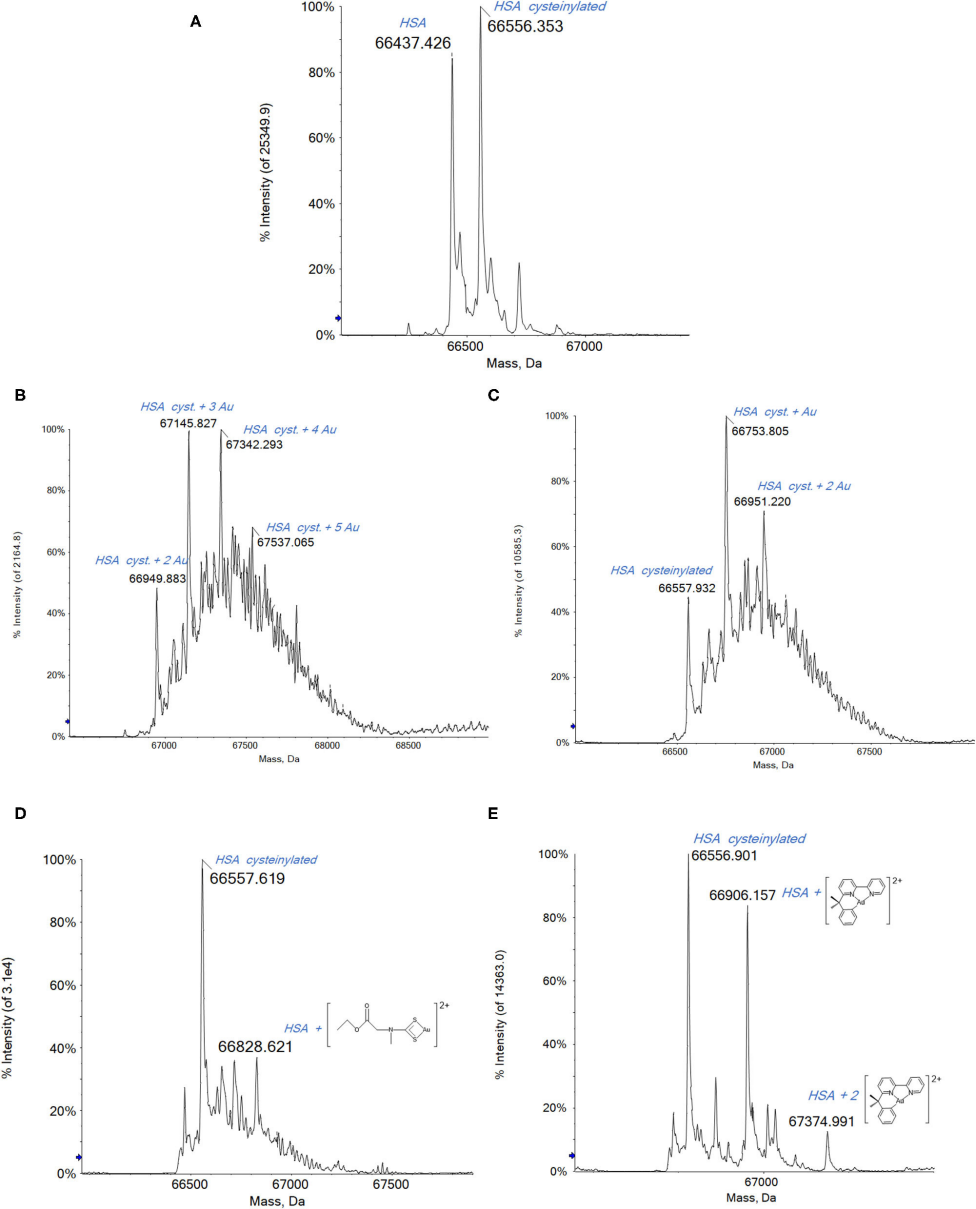
**(A)** Deconvoluted electrospray ionization quadrupole time-of-flight (ESI-Q-TOF) spectra of human serum albumin (HSA) 5 × 10^−7^ M in 2 mM of ammonium acetate solution at pH 6.8 and incubated at 37 °C for 2 h with **(B)** Auoxo6, **(C)** Au_2_phen, **(D)** AuL12, and **(E)** Aubipyc solution in dimethyl sulfoxide (DMSO) in a 1:0.9 protein-to-gold ratio; 0.1% v/v of formic acid was added just before infusion.

The deconvoluted high-resolution mass spectrum of HSA is shown in Figure 2A. The main peak at 66,437 Da corresponds to the native protein and the second intense peak at 66,556 Da is assigned to a common HSA posttranslational modification, i.e., the cysteinylated form of the native protein (Fasano et al., 2005; Talib et al., 2006; Fanali et al., 2012).

Upon reacting HSA with either Auxo6 or Au_2_phen, an apparent decrease in the overall quality of the ESI MS spectra is noticed, which is accompanied by a strong baseline distortion, with both tested stoichiometries (1:3 or 1:0.9 protein/metal ratio). In our previous experience, this behavior is quite common when dealing with metal complexes where the metal center is present in its higher oxidation state; probably this arises from the occurrence of a direct redox reaction between the metal complex and the biomolecule, leading to a plethora of minor adducts that are present in small amounts and are difficult to assign (Massai et al., 2019; Pratesi et al., 2019).

In spite of that, the resulting spectra still allow the detection of a few metal–protein adducts with greater molecular masses that the native protein. The peaks corresponding to these adducts are annotated on the spectra shown in Figure 2. In the case of Auoxo6 and Au_2_Phen (Figures 2B,C, respectively), the gold adducts are formed on the cysteinylated protein. These adducts are characterized by the probable presence of gold clusters, which in the case of the most reactive Auoxo6 (Figure 2B) complex consist of a maximum of 5 gold atoms, represented by the peak at 67,537 Da. Also in the case of Au_2_Phen, the adducts are formed on the cysteinylated HSA, but due to the lower reactivity of Au_2_Phen in comparison with Auoxo6, the gold clusters formed on the protein consist of a maximum of 2 gold atoms, with a total molecular mass of 66,951 Da (Figure 2C). A further confirmation of the presence of metallic gold clusters can be obtained from the inspection of the multicharged ESI spectra. In fact, the presence of a metal cluster, differently from the metal ions, does not bring any contribution to the overall charge of the adducts and does not affect the *m*/*z* ratio of the multicharged signals that is due in this case only to the protonation process inside the ESI source (Johnson et al., 2000; Johnson and Laskin, 2016; Chen et al., 2019).

Far better spectra are obtained upon reacting HSA with AuL12 (Figure 2D). Remarkably, the peak observed at 66,779 Da corresponds to a species where the Au(III) ethylsarcosine dithiocarbamate (ESDT) fragment is bound to the protein. Additional peaks at lower mass values are seen corresponding to the Au(I)/HSA adduct, implying that AuL12 may also undergo reduction to gold(I) with loss of the dithiocarbamate ligand, as already described in a previous work of ours concerning the reactivity of the bovine serum albumin (BSA) with AuL12 (Pratesi et al., 2019).

Contrariwise, the Aubipyc complex is far more stable from the redox point of view than the previously mentioned gold(III) compounds (Gabbiani et al., 2012b; Marzo et al., 2015; Kupiec et al., 2019). Usually, the reduction of the gold(III) center is not observed for this complex. It follows that upon reaction with Aubipyc, the spectrum of HSA is dominated by a peak at 66,906 Da corresponding to the native HSA bearing the Aubipyc moiety without the hydroxo group (Figure 2E). Another peak at 67,394 Da corresponds to the bis-adduct of HSA with the same molecular fragment. In contrast with the results described for the previous gold(III) compounds, in this case, the lack of adducts formed with cysteinylated HSA suggests that this modification of the only free cysteine prevents the reaction with Aubipyc. This could be due to the greater stability of the –S–S– bond in the cysteinylated HSA with respect to the potential –S–Au(III) interaction with the Aubipyc reactive moiety. Thus, a clearly different behavior has been highlighted for the Aubipyc complex compared with Auoxo6 and Au_2_phen.

### The Reactions of Gold Compounds With Human Carbonic Anhydrase I

On the whole, the ESI MS spectra obtained for hCA I and its adducts with the study gold compounds turned out to be of greater quality than those of HSA, possibly in relation to the greater ionizability of this protein and its lower size.

Human carbonic anhydrase I (hCA I) consists of 261 amino acids and contains a Zn(II) ion essential for catalysis. The enzyme presents a free cysteine (Cys213) as a potential anchoring site for gold.

**Figure 3:**
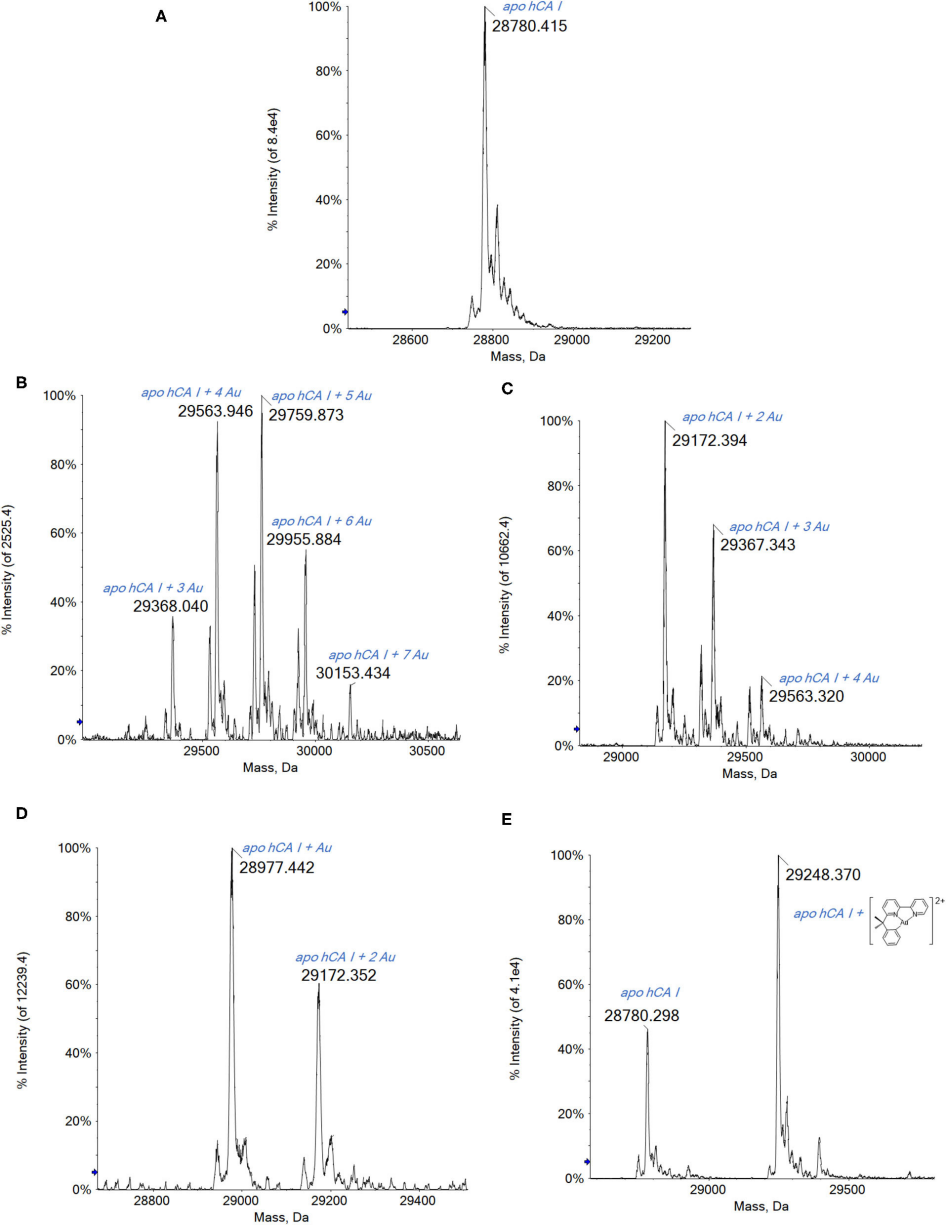
**(A)** Deconvoluted electrospray ionization quadrupole time-of-flight (ESI-Q-TOF) spectra of human carbonic anhydrase I (hCA I) 7 × 10^−7^ M in 2 mM of ammonium acetate solution at pH 6.8 and incubated at 37 °C for 2 h with **(B)** Auoxo6, **(C)** Au_2_phen, **(D)** AuL12, and **(E)** Aubipyc solution in dimethyl sulfoxide (DMSO) in a 1:0.9 protein-to-gold ratio; 0.1% v/v of formic acid was added just before infusion.

The analysis of the ESI MS spectra is quite straightforward. Indeed, when reacting hCA I with the three gold(III) complexes endowed with a more oxidizing character, i.e., Auoxo6, Au_2_phen, and AuL12, adducts with an increasing number of Au atoms bound to apo-hCA I are again detected (Figure 3).

Regarding the complex AuL12, the recorded spectra show the formation of the mono- and bis-adduct with the Au atoms (Figure 3D).

Noteworthy, the ESI MS spectra of hCA I reacted with Auoxo6 or Au_2_Phen show many signals that are attributed to the binding to hCA I of a number of Au atoms in different stoichiometries: from 3 to 7 in the case of Auoxo6 (Figure 3B) and from 2 to 4 for Au_2_Phen (Figure 3C). Moreover, it is worth noting that in both spectra (Figures 3B,C), the signal of unreacted apo-hCA I (28,780 Da) is not observed any more, meaning that the reaction with the gold compounds is rapid and nearly quantitative.

Thus, in the cases of Auoxo6, Au_2_Phen, and AuL12, a gold(III)-to-gold(0) reduction reaction occurs accompanied by the release of all the original gold(III) ligands. Also in this case, an inspection of the multicharged ESI spectra has been carried out to confirm the presence of gold clusters. Again, Aubipyc reveals a different behavior with no evident reduction of the gold(III) center and retention of the terdentate ligand. Only one Aubipyc moiety is found bonded to hCA I in the main adduct detected at 29,248 Da (Figure 3E).

### The Reactions of Gold Compounds With the C-Terminal Dodecapeptide of TrxR1

Finally, the four gold(III) complexes were challenged with the C-terminal dodecapeptide of TrxR bearing the –Cys–Sec– motif. This peptide has been adopted since a few years by our research group as a model peptide mimicking the TrxR1 reactive site (Pratesi et al., 2010, 2014).

This peptide is characterized by the presence of an intramolecular –S–Se– bridge that requires reduction to give the reactive form of the peptide. This reaction is achieved by adding 10 eq. of dithiothreitol (DTT) before the incubation with the various gold complexes (Pratesi et al., 2014). The presence of the reductant greatly facilitates the reaction between selenocysteine and cysteine with the gold complexes.

**Figure 4:**
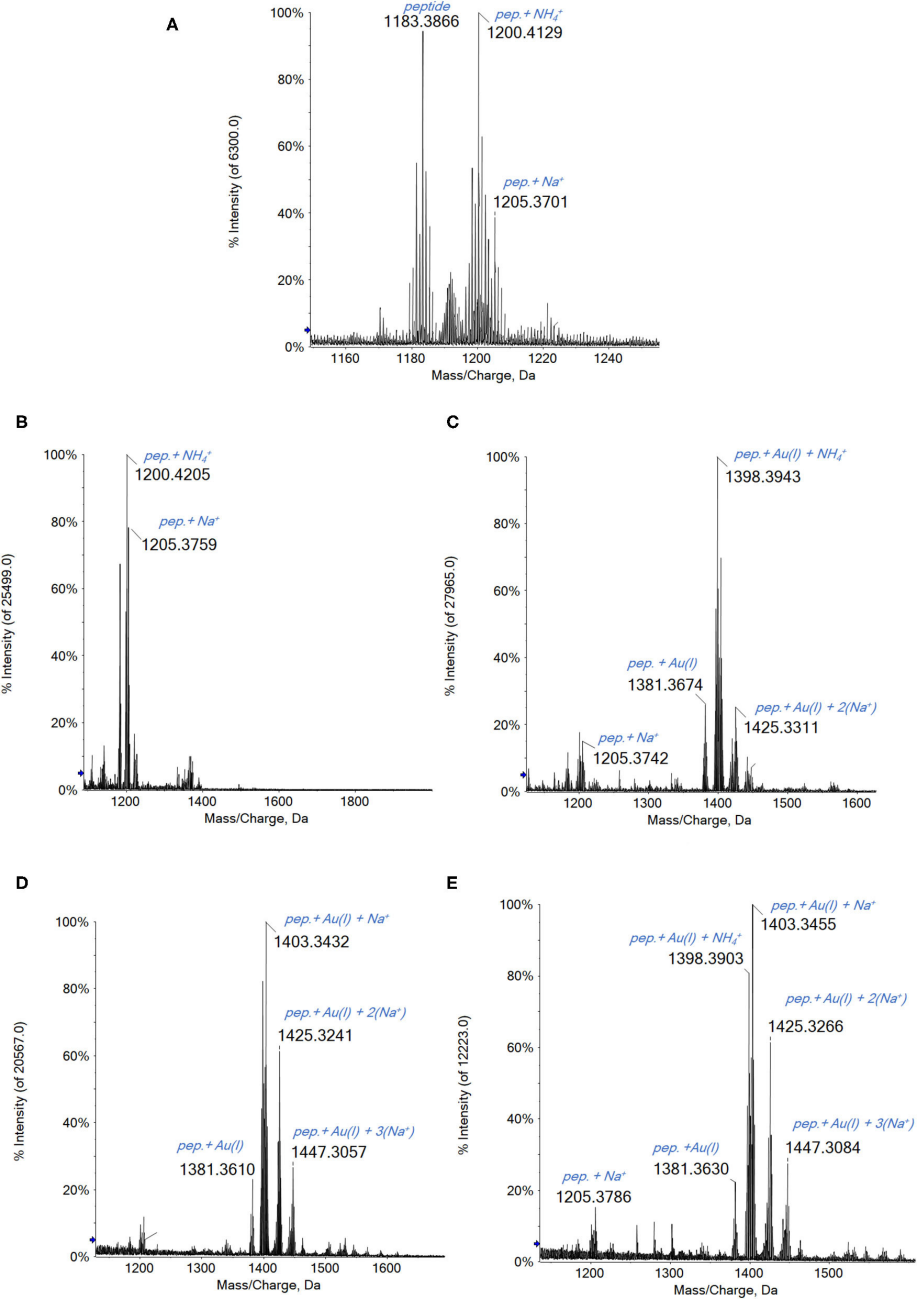
**(A)** Multicharged electrospray ionization quadrupole time-of-flight (ESI-Q-TOF) spectra of the dodecapeptide 5 × 10^−7^ M in 2 mM of ammonium acetate solution at pH 6.8 and incubated at 37 °C for 2 h with **(B)** Auoxo6, **(C)** Au_2_phen, **(D)** AuL12, and **(E)** Aubipyc solution in dimethyl sulfoxide (DMSO) in a 1:3:5 protein-to-gold-to-DTT ratio; 0.1% v/v of formic acid was added just before infusion.

A general reaction pattern is identified also in this case being perfectly superimposable to that of the other considered proteins. In particular, upon reacting Au_2_phen, AuL12, and even Aubipyc with the dodecapeptide, the main adduct formed corresponds to the binding of a gold(I) ion to the peptide (Figure 4). In addition, other minor signals are also present, related to adducts with higher Au(I)/peptide ratios. Apparently, in the case of Auoxo6, no reaction is observed under the same experimental conditions.

Interestingly, in the case of this peptide, the gold compounds' reactivity is quite similar to that observed with the proteins. The main difference is that, in this latter case, the complete reduction from Au(III) to Au(0) does not occur. This behavior could be certainly ascribed to the marked difference in the chemical environment of the whole protein compared with a peptide fragment; however, this aspect will be further investigated.

Since Auoxo6 is the only compound that shows no reactivity with the peptide after a short time of incubation (2 h), other experiments were conducted. Different concentration ratios between the three reagents (the peptide, Auoxo6, and the DTT) were tested, in order to find the one that allows the adduct formation in short times. The best ratio was found to be the 1:1:10 (peptide to gold to DTT) in which a certain reactivity of the gold compound is observed even at short times of incubation. Indeed, after 2 h, the signal at 1,398 Da attributed to the adduct of the peptide with Au(I) (and a ${\text{NH}}_{4}^{+}$ ion from the solution) is clearly observed (see Supplementary Figure 11), even if the signals of the peptide alone are still quite intense. After 24 h of incubation, no adduct signal is detected anymore.

### The Reactivity of Aubipyc in the Presence of a Reducing Agent

The above-described experiments highlighted a different behavior of Aubipyc depending on the substrate considered. Indeed, upon reaction with HSA and hCA I, Aubipyc binds these proteins through the [Au(III)(bipydmb-H)]^2+^ fragment, with the gold center retaining the oxidation state +3; in contrast, upon reaction with the dodecapeptide, Aubipyc undergoes complete reduction and loss of the ligand so that an adduct containing a single Au(I) ion is formed.

When performing the experiments with the peptide, a reducing agent, i.e., DTT, is needed to break the bond between the cysteine and selenocysteine residue and promote the binding of the metal. In order to assess if the reduction of the gold center in Aubipyc is due to DTT or, instead, to the biological substrate, an additional experiment was conducted. A sample of HSA was prepared with Aubipyc and DTT in a ratio of 1:0.9:5 of protein to gold to reducing agent. After 2 and 24 h of incubation at 37 °C, the mass spectra (see Supplementary Figure 1) reveal the presence of the intense signal at 66,906 Da attributed to the native protein binding the [Au(III)(bipydmb-H)]^2+^ moiety. Though less intense, the cysteinylated HSA signal is still present. So the spectra are identical to those registered in the absence of DTT (Figure 4). Therefore, despite the presence of an excess of a reducing agent like DTT, Aubipyc when reacting with HSA retains the ligand and the oxidation state of +3. So it can be inferred that the reduction of the gold center to gold(I) in the case of the C-terminal dodecapeptide of TrxR is due to the nature of the substrate and not to the presence of DTT.

## Complementary Biophysical Measurements

To gain more information about the interaction of the selected gold(III) complexes with the three targets, other experiments were carried out. More precisely, spectrophotometric and ^1^H NMR measurements were performed for achieving, if possible, independent confirmations of the results obtained through ESI MS measurements. In some cases, additional and valuable information was gained.

### Spectrophotometric Measurements

**Figure 5:**
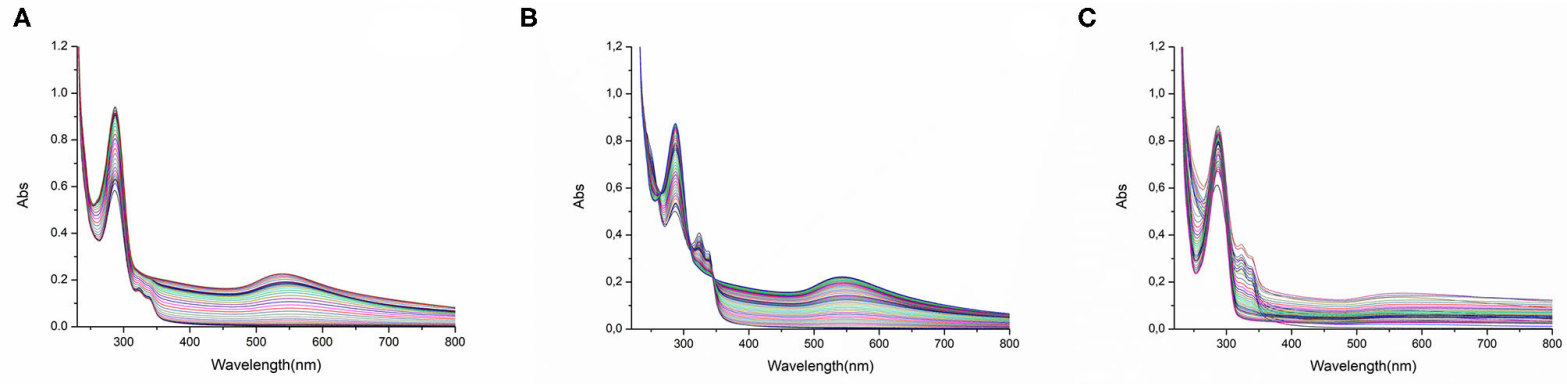
UV–Vis spectra of Auoxo6 with **(A)** HSA, **(B)** hCA I and **(C)** the dodecapeptide 10^−5^ M in phosphate buffer 50 mM, pH 7.4 (1:3 biomolecule-to-gold ratio).

In the case of Auoxo6, it was possible to elucidate a common redox-dependent binding process found for the interaction with all the three targets used in this investigation (Figure 5). More precisely, the time course spectra of samples offer a clear evidence of the occurrence of a gold(III) reduction mechanism, highlighted from the disappearance of the complex absorption bands located between 310 and 330 nm. Moreover, the reduction of Auoxo6 signals is accompanied by the growth of a new band located at 290 nm and assignable to the free 6,6′-dimethyl-2,2′-bipyridyl ligand. In the case of HSA and hCA I (Figures 5A,B), in addition, a new relevantly broad band appears in the visible region between 500 and 600 nm, usually diagnostic of the formation of elemental gold clusters (Dominguez-Medina et al., 2012; Tatini et al., 2014). Notably, all these results turned out to be in perfect agreement with those observed through the ESI MS experiments, in which HSA and hCA I show the binding with a polynuclear gold(0) cluster upon the interaction of Auoxo6, contrary to the case of thioredoxin reductase dodecapeptide model that was found to bind a naked gold(I) atom.

Also in the case of Au_2_Phen, it was possible to gain a clear evidence of gold(0) formation at least in the case of the interaction with hCA I, confirming again the data collected through MS; on the other hand, Aubipyc, as already emerged from ESI MS measurements, does not seem to incur to any reduction process (see Supplementary Material for detailed spectra of these latter data: Supplementary Figures 7–10, 12, 13).

### ^1^H Nuclear Magnetic Resonance Measurements

In order to confirm the reaction pattern observed in the ESI MS experiments, some NMR measurements were carried out. First of all, each investigated complex was incubated for 24 h with HSA. Subsequently, ^1^HNMR spectra were acquired through a Carr–Purcell–Meiboom–Gill pulse sequence used as a T2 filter for removing the HSA signals. Indeed, this experimental procedure is used for achieving the suppression of all resonances with short T2, typically belonging to high-molecular-weight molecules such as HSA or HSA/metal–complex adducts, allowing to record spectra not affected from broad signals that might hide small trace of complexes' degradation products (Pratesi et al., 2019). The NMR experiments turned out to be nicely in agreement with the ESI MS study.

**Figure 6:**
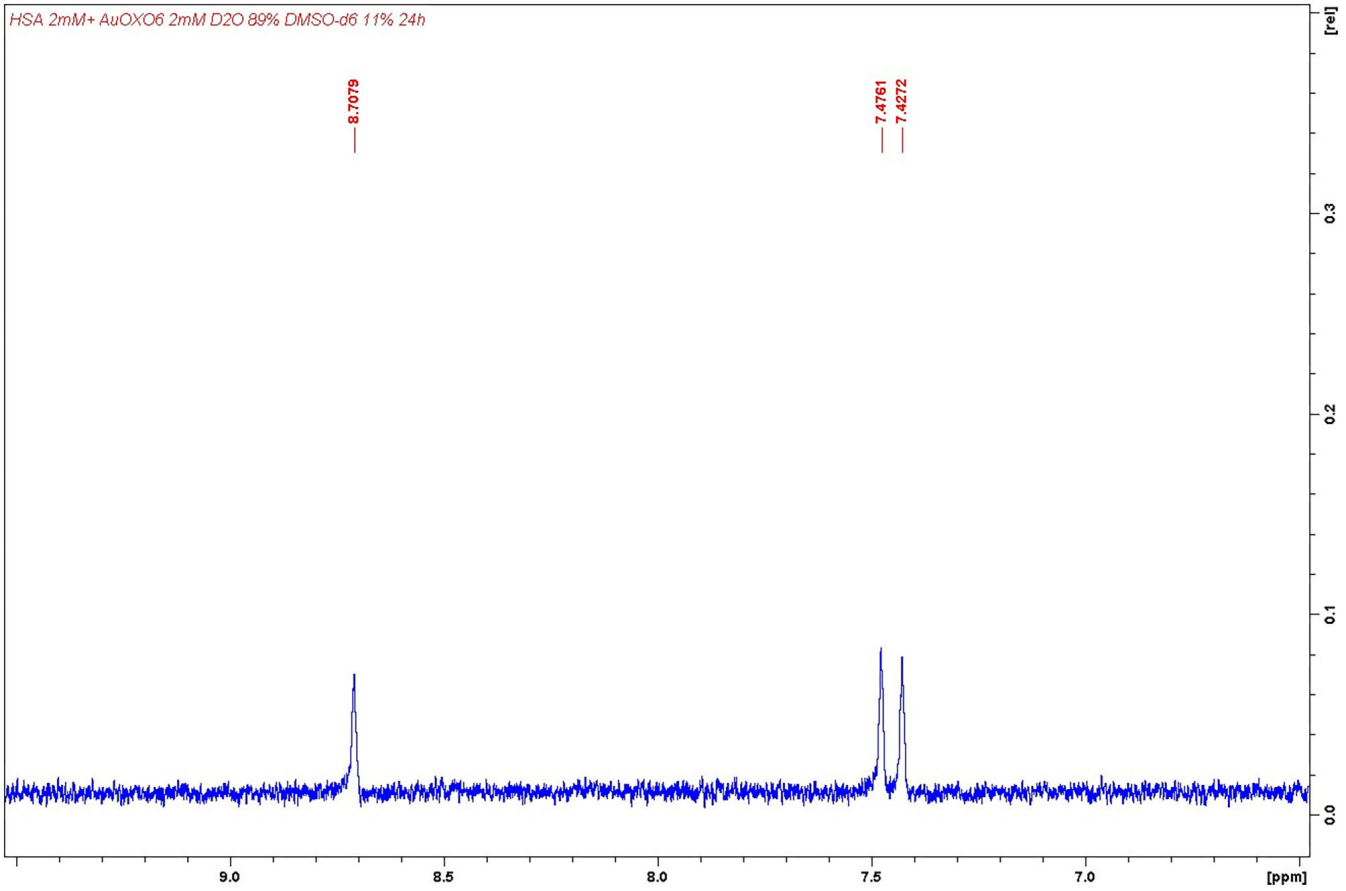
Detail of 6,6′-dimethyl-2,2′-bipyridyl ^1^HNMR aromatic signals. Detection performed after Auoxo6 interaction with human serum albumin (HSA).

More precisely:

- Upon incubation with HSA, AuL12 shows the same fragmentation pattern seen upon interaction with BSA (Pratesi et al., 2019). Indeed, also in this case, it is possible to identify two small molecules originated from AuL12 degradation mechanisms, i.e., ethanol (δ 3.64, 1.17) and ethyl sarcosinate (δ 4.30, 3.96, 2.79, 1.29) (see Supplementary Figure 5). This occurrence is in agreement with ESI MS data, in which only a gold/HSA adduct is visible in the spectrum, with a complete loss of the ligand from the metal center.
- Contrary to the previous case, no Aubipyc signals were detected after incubation with HSA. The fact that all its resonances were completely suppressed from the T2 filter upon HSA interaction can easily be explained by assuming a coordination process on the protein scaffold in which the organic moiety of the metal complex was retained on the gold center. This latter occurrence was again in agreement with ESI MS experiments.
- In the case of Auoxo6, signals imputable to 6,6′-dimethyl-2,2′-bipyridyl ligand were detected in solution as a consequence of the degradation of the complex (δ 8.71, 7.48, 7.43, one proton each; δ 3.34, three protons from the methyl group); see Figure 6. This latter result was again in perfect agreement with that obtained through ESI MS measurements, in which only a gold/HSA adduct was found.

The case of the interaction of Au_2_phen with HSA turned out to be slightly more complicated. More precisely, the spectrum of the sample showed no signals, suggesting a behavior of Au_2_phen similar to that of Aubipyc. On the other hand, this occurrence seems to be in contrast with ESI MS results, in which only the presence of the same gold/HSA adduct was detected as in the case of interactions with AuL12 and Auoxo6. Anyway, this apparent contradiction can easily be explained, taking into account the precipitation of the ligand during the incubation process. Indeed, after the mixing of HSA and Au_2_phen, the formation of a precipitate occurs, most probably an adduct between HSA and the aromatic ligand lost from Au_2_phen. On the other hand, the formation of adducts between HSA and 1,10-phenanthroline systems was already reported in literature (Lin and Shen, 2017).

## Conclusions—A General Interpretation of the Obtained Results

The above-reported results permit to identify some characteristic trends in the reactivity of the study gold(III) compounds with the tested protein targets. In particular, MS results provide a substantial body of comparative data on the various investigated systems. From the precise determination of the formed adducts, inferences may be drawn on the type of the occurring reactions in relation to the nature of the metal complex and of the interacting biomolecule.

From careful data analysis, some conclusions may be extracted, as follows:

The reactivity pattern first depends on the specific nature of the metal complex and its oxidizing character. Indeed, the more oxidizing gold(III) agents invariantly tend to undergo reduction to gold(I) or gold(0); the reduction process is generally associated with complete metal complex degradation, with the exception of AuL12, which retains the dithiocarbamate ligand. Typically, the less-oxidizing Aubipyc in its reaction with HSA and hCA I tends to conserve the gold center in the oxidation state +3 with complete retention of the terdentate ligand. Also, a not-negligible role is played by the nature of the protein. Indeed, the C-terminal dodecapeptide of thioredoxin reductase was found to be able to induce reduction even of the Aubipyc complex to gold(I) with the loss of the ligand.

In conclusion, from these results, the prodrug character of these metal complexes is further documented. All of them are able to be a source of gold(I) ions that are capable of metalating proteins mostly at cysteine and selenocysteine residues. Production of gold(I) ions may be modulated through the oxidizing character of the complexes, especially for Auoxo6 and Au2phen, and the kinetics of the reduction reaction. Regarding Aubipyc, the different biomolecules promote the formation of different binding moieties; indeed, in the case of HSA and hCA I, the metal complex retains its oxidation state (+3), and the [Au(III)(bipy^dmb^-H)]^2+^ fragment binds the proteins, while in the presence of the dodecapeptide, a reduction of the gold center occurs, and the formation of Au(I)–peptide adduct is revealed.

Remarkably, in the presence of an excess of the gold complex, formation of small clusters containing 2–7 atoms of elemental gold is observed.

As indicated in several papers (Magherini et al., 2010; Cattaruzza et al., 2011; Huang et al., 2018; Altaf et al., 2019), the mechanisms of action of these gold(III) compounds seem to rely ultimately on protein metalation; moreover, the oxidizing character of the starting complex may play a role in the overall cytotoxic mechanism, further exacerbating the intracellular oxidative stress and reactive oxygen species (ROS) generation. The results of our work, highlighting the behavior of the gold(III) complexes in the presence of different biomolecules, seem to be an additional evidence to support the importance of redox mechanisms in the biological activity of these compounds.

## Materials and Methods

### Materials

Lyophilized hCA I and HSA were purchased from Sigma-Aldrich and used without further purification or manipulation. Auoxo6, Au_2_phen, Aubipyc, and AuL12, as well as the dodecapeptide of thioredoxin reductase (dTrxR), were synthetized in the MetMed Laboratories at the Department of Chemistry, University of Florence, following already established procedures (Pratesi et al., 2010, 2014).

DTT and dimethyl sulfoxide (DMSO) were purchased from Fluka. Liquid chromatography (LC)–MS materials (water, methanol, and ammonium acetate) were purchased from Sigma-Aldrich. Deuterated solvents (D_2_O and DMSO-*d*_6_) were purchased from Sigma-Aldrich.

### Electrospray Ionization Mass Spectrometry Experimental Conditions

#### Sample Preparation

Stock solutions of hCA I 10^−4^ M, HSA 10^−3^ M, and dTrxR 10^−3^ M were prepared, dissolving the proteins and the peptide in H_2_O of LC-MS grade. Stock solutions 10^−2^ M of the gold(III) compounds were prepared by dissolving the samples in DMSO. Stock solution 10^−1^ M of DTT was prepared in H_2_O.

For the experiments with HSA, solutions of the protein 10^−4^ M and each gold compound at protein-to-metal ratios 1:0.9 or 1:3 were prepared by diluting with ammonium acetate solution 2 × 10^−3^ M, pH 6.8. The mixtures were then incubated at 37 °C up to 24 h.

For Aubipyc, another sample was prepared, as follows: a solution 10^−4^ M of the protein was prepared by diluting with ammonium acetate solution 2 × 10^−3^ M, pH 6.8. Then an aliquot of DTT stock solution was added in a protein/reducing agent 1:5 ratio; then the mixture was incubated at 37 °C up to 30 min. After that time, an aliquot of Aubipyc was added in a protein-to-metal compound ratio of 1:0.9. The mixture thus obtained was incubated at 37 °C up to 24 h.

For the experiments with hCA I, solutions of the protein 10^−5^ M and each gold compound at protein-to-metal ratio 1:3 were prepared by diluting with ammonium acetate solution 2 × 10^−3^ M, pH 6.8. The mixtures were then incubated at 37 °C up to 24 h.

For the experiments with TrxR peptide, solutions of the peptide 10^−4^ M were prepared by diluting with ammonium acetate solution 2 × 10^−3^ M, pH 6.8. Then aliquots of DTT stock solution were added in a peptide to reducing agent ratios (1:5, 1:10); then the mixtures were incubated at 37 °C up to 30 min. After that time, aliquots of the gold(III) compounds' solutions were added in peptide-to-metal compound ratios 1:1 or 1:3. The mixtures thus obtained were incubated at 37 °C up to 24 h.

#### Electrospray Ionization Mass Spectrometry Analysis: Final Dilutions

After the incubation time, all solutions were sampled and diluted to a final protein concentration of 7 × 10^−7^ M for hCA I, 5 × 10^−7^ M for HSA, and dTrxR using ammonium acetate solution 2 × 10^−3^ M, pH 6.8.

The HSA and hCA I final solutions were also added with 0.1% v/v of formic acid just before the infusion in the mass spectrometer.

#### Instrumental Parameters

The ESI mass study was performed using a TripleTOF® 5600^+^ high-resolution mass spectrometer (Sciex, Framingham, MA, United States), equipped with a DuoSpray® interface operating with an ESI probe. Respective ESI MS spectra were acquired through direct infusion at 5 μl/min of flow rate.

The general ESI source parameters optimized for each protein and peptide analysis were as follows:

HSA: positive polarity, ion spray voltage floating 5,500 V, temperature 0, ion source Gas 1 (GS1) 45 L/min; ion source Gas 2 (GS2) 0; curtain gas (CUR) 12 L/min, collision energy (CE) 10 V; declustering potential (DP) 150 V, range 1,000–2,600 *m*/*z*.

hCA I: positive polarity, ion spray voltage floating 5,500 V, temperature 0, ion source Gas 1 (GS1) 40 L/min; ion source Gas 2 (GS2) 0; CUR 10 L/min, CE 10 V; DP 50 V, range 760–990 *m*/*z*.

dTrxR: positive polarity, ion spray voltage floating 5,500 V, temperature 100 °C, ion source Gas 1 (GS1) 25 L/min; ion source Gas 2 (GS2) 25 L/min; CUR 30 L/min, CE 10 V; DP 50 V, range 1,000–2,000 *m*/*z*.

For acquisition, Analyst TF software 1.7.1 (Sciex) was used, and deconvoluted spectra were obtained by using the Bio Tool Kit micro-application v.2.2 embedded in PeakView™ software v.2.2 (Sciex).

### Nuclear Magnetic Resonance

Samples were prepared as an HSA solution in D_2_O in which DMSO-*d*_6_ stock solutions of the investigated compounds were added. The final concentrations were 2 mM of HSA with 1 equivalent of gold compound in D_2_O/DMSO-*d*_6_ 9:1. Samples were then incubated for 24 h at 37 °C before acquisition.

Acquisitions were performed using cpmg1dpr Bruker pulse sequence (monodimensional Carr–Purcell–Meiboom–Gill sequence with solvent presaturation). Offset frequency was set up to 4.79 ppm for achieving residual H_2_O suppression, while echo time was set up to 6 ms and the echo loop repeated for 126 times before free induction decay (FID) acquisition (1.512 s of total delay).

### UV–Visible Spectrophotometric Studies

The electronic spectra were recorded by diluting small amounts of freshly prepared concentrated solutions of HSA, hCA I, or dodecapeptide, in the reference solution (2 mM of ammonium acetate, pH 6.8), at final protein/peptide concentration of 10^−5^ M. Each gold complex has been added, after the recording of the protein/peptide baseline, at a stoichiometric ratio of 3:1 (metal to protein). The resulting solutions were monitored by collecting the electronic spectra over 24 h at 37 °C.

## Data Availability Statement

All datasets generated for this study are included in the article/Supplementary Material.

## Author Contributions

AP, LMa, and LMe designed, coordinated, and supervised the whole study. DC carried out the NMR experiments. CZ and LMa acquired all the ESI MS and UV–Vis spectra. AP, DC, and LMe wrote the manuscript. All authors revised the experimental part and the discussion and approved the final version of the manuscript. All authors contributed to the article and approved the submitted version.

## Conflict of Interest

The authors declare that the research was conducted in the absence of any commercial or financial relationships that could be construed as a potential conflict of interest.
