# Supplementary material for: Reactions of Medicinal Gold(III) Compounds With Proteins and Peptides Explored by Electrospray Ionization Mass Spectrometry and Complementary Biophysical Methods
Source: Front Chem. 2020 Oct 21;8:581648. doi: 10.3389/fchem.2020.581648 (PMC7609534; doi:10.3389/fchem.2020.581648)
Supplement: Supplementary file 1 [file Data_Sheet_1.PDF]

Supplementary Material

Reactions of Medicinal Gold(III) Compounds with Proteins and Peptides Explored by ESI MS and complementary Biophysical Methods.

Lara Massai, Carlotta Zoppi, Damiano Cirri, Alessandro Pratesi*, and Luigi Messori*

Index

1 HSA 2
2 hCA I 10
3 Peptide fragment of TrxR 12

1 HSA

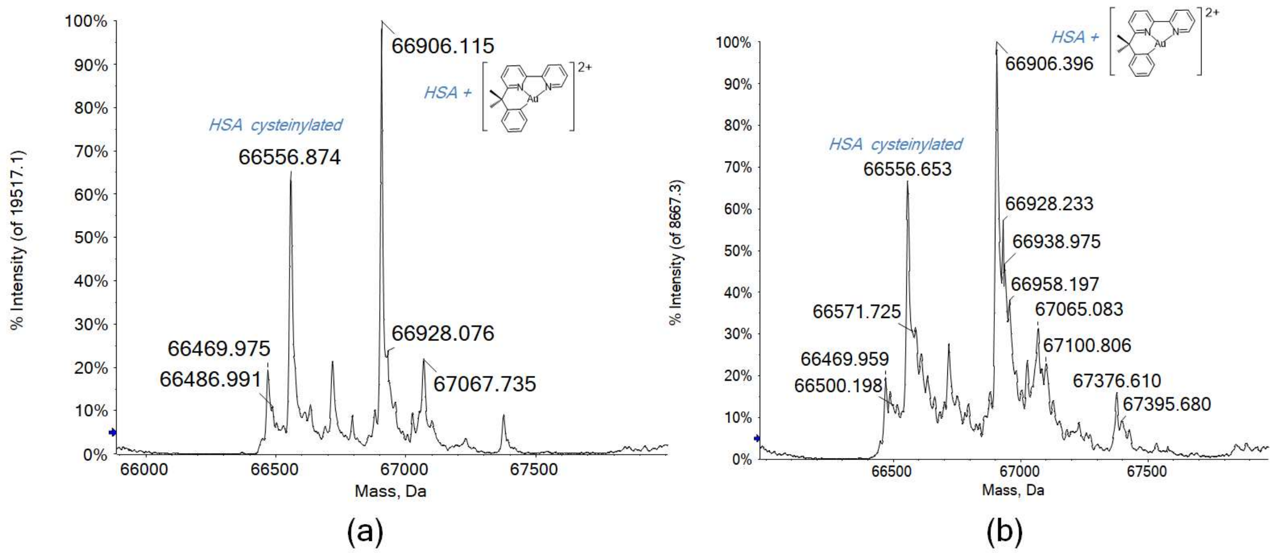

Supplementary Figure 1. Deconvoluted ESI Q TOF spectrum of HSA solution 5x10^{-7} M with DTT and Aubipyc (1:5:0.9 protein/reducing agent/gold ratio) in ammonium acetate solution 2 mM (pH 6.8) after a) 2 h and b) 24 h of incubation at 37 °C

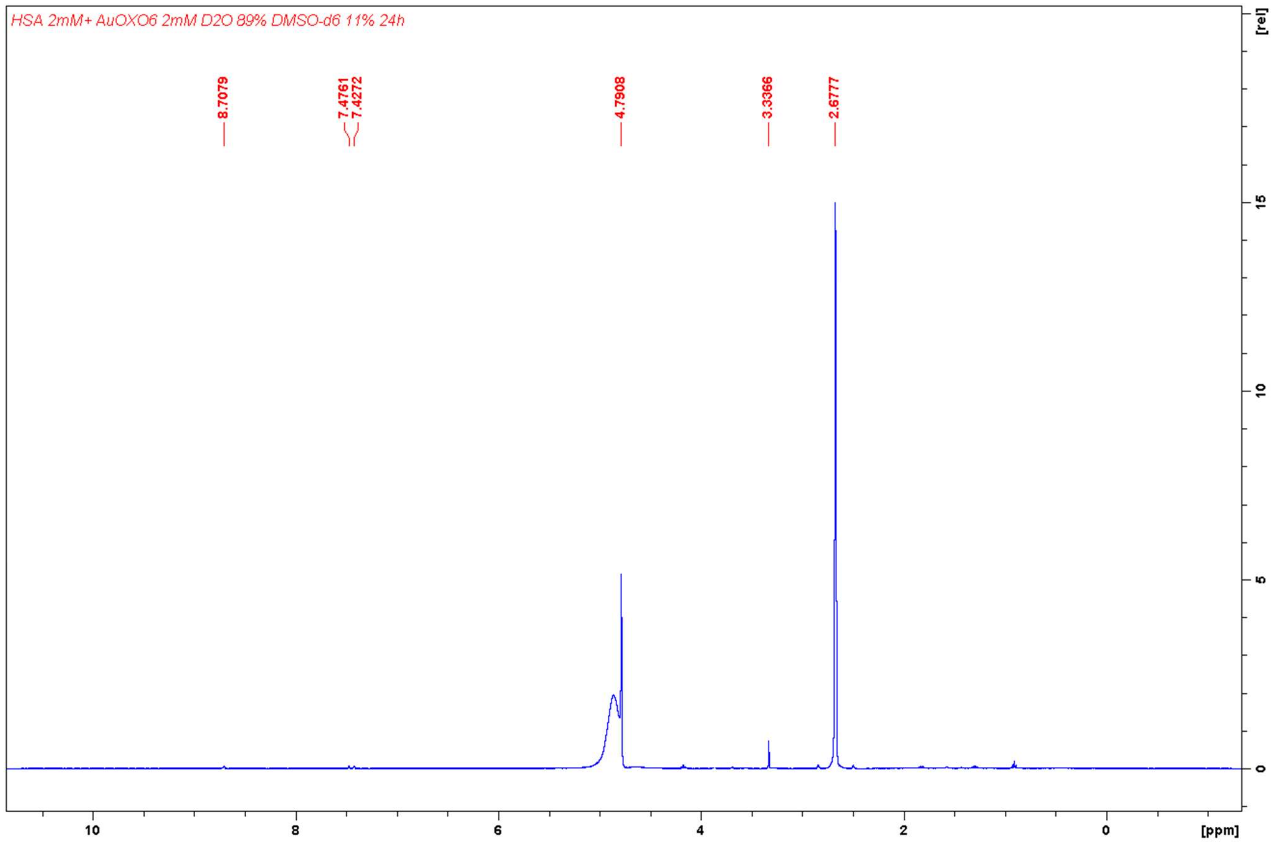

Supplementary Figure 2. ^{1}HNMR spectrum of HSA solution 2 mM with Auoxo6 (1:1 protein/gold ratio) in D_2O/DMSO-d_6 9:1 after 24 h of incubation at 37 °C.

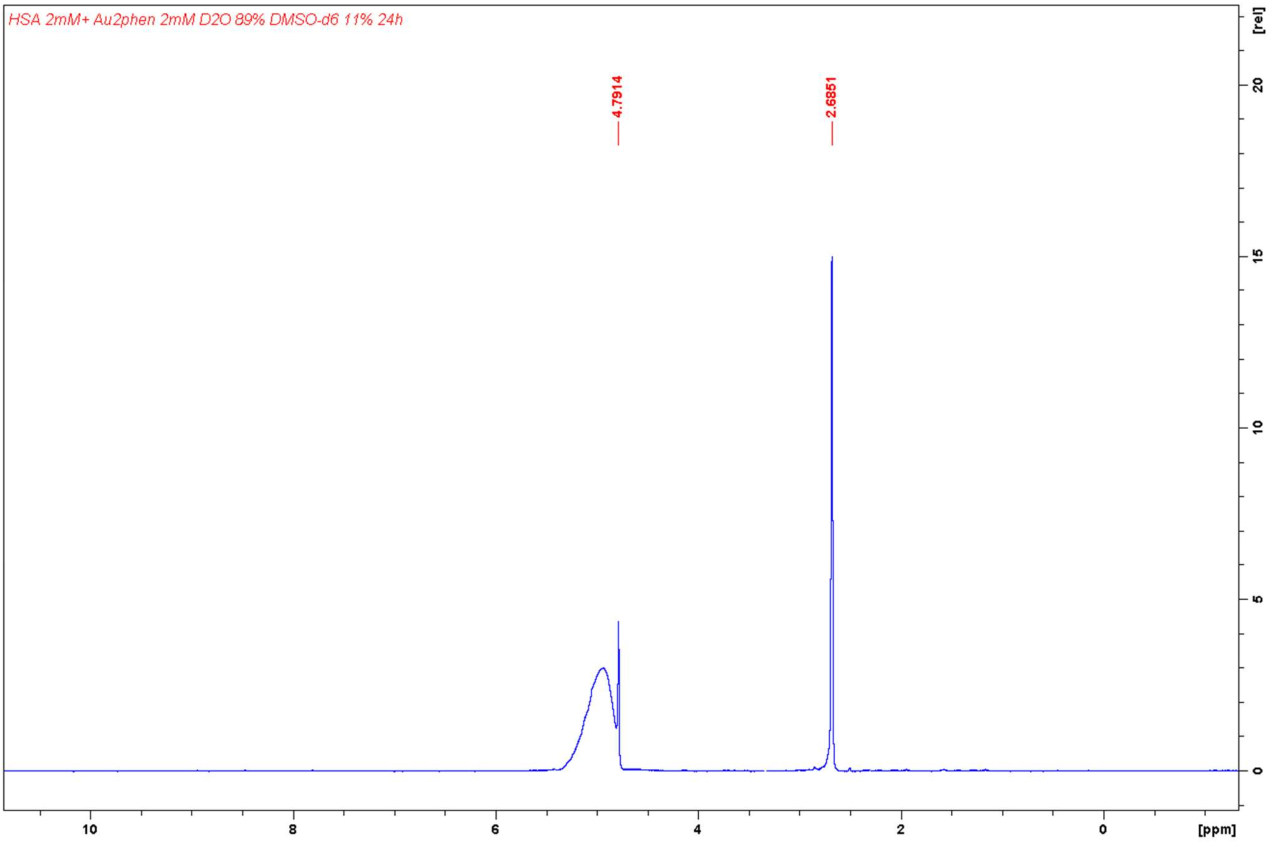

Supplementary Figure 3. ^{1}HNMR spectrum of HSA solution 2 mM with Au_2Phen (1:1 protein/gold ratio) in D_2O/DMSO-d_6 9:1 after 24 h of incubation at 37 °C.

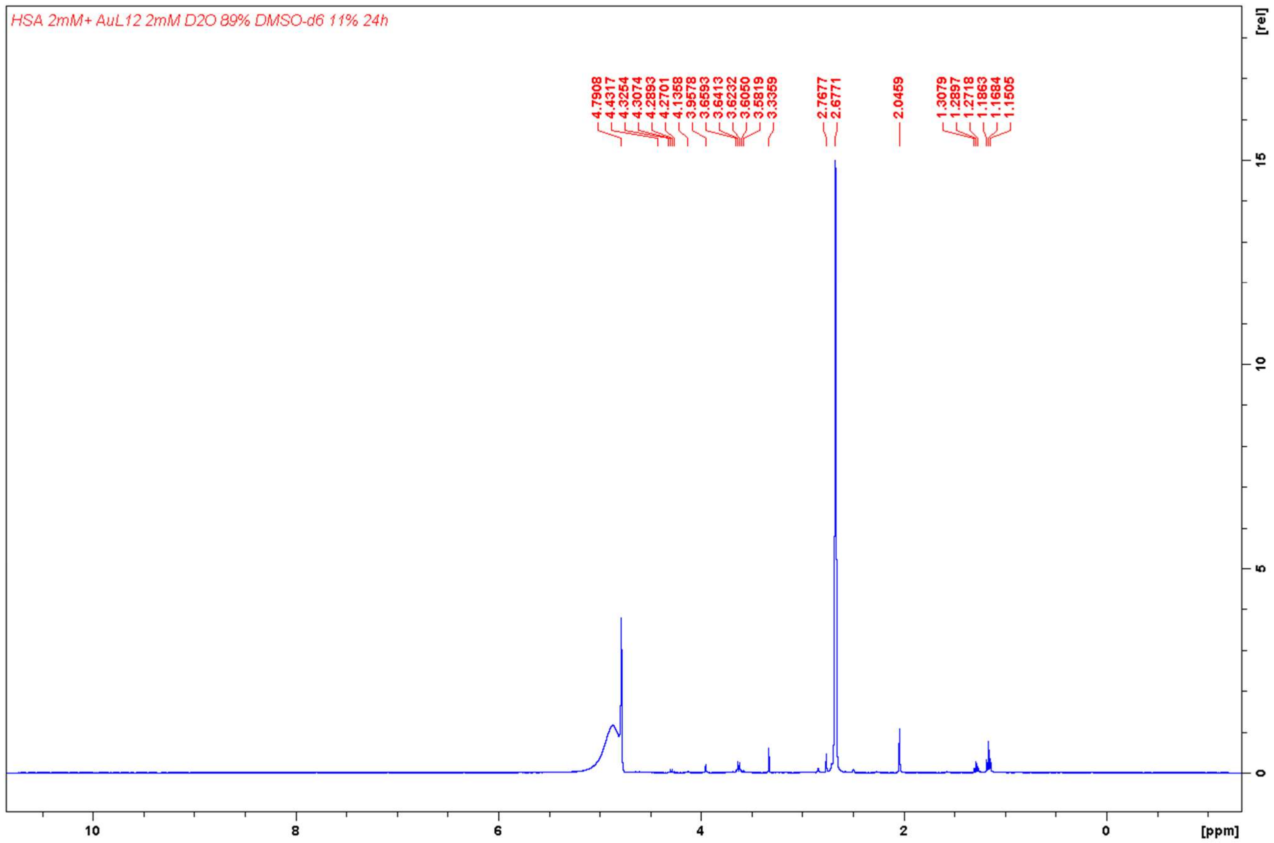

Supplementary Figure 4. ^{1}HNMR spectrum of HSA solution 2 mM with AuL12 (1:1 protein/gold ratio) in D_2O/DMSO-d_6 9:1 after 24 h of incubation at 37 °C.

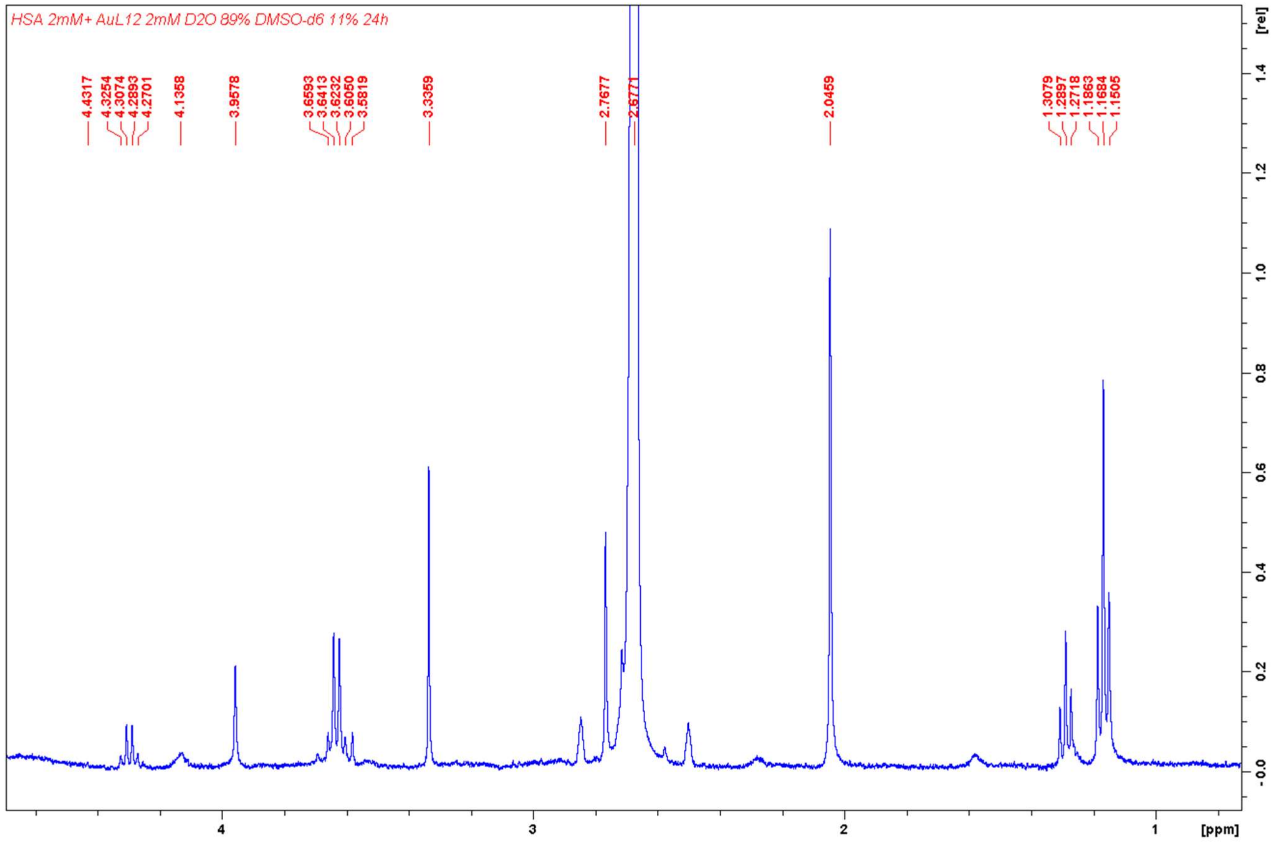

Supplementary Figure 5. ^{1}HNMR spectrum of HSA solution 2 mM with AuL12 (1:1 protein/gold ratio) in D_2O/DMSO-d_6 9:1 after 24 h of incubation at 37 °C. Zoom of the alifatic section.

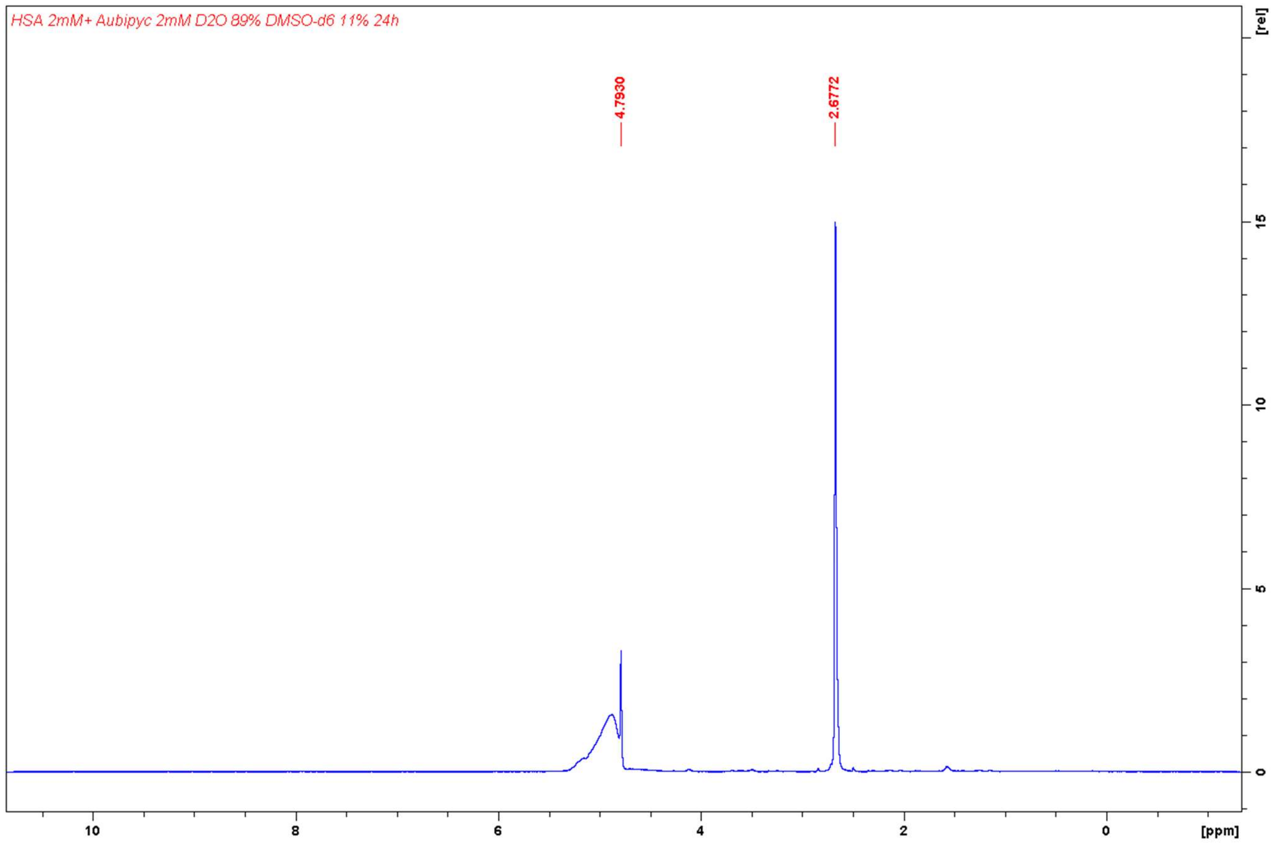

Supplementary Figure 6. ^{1}HNMR spectrum of HSA solution 2 mM with Aubipyc (1:1 protein/gold ratio) in D_2O/DMSO-d_6 9:1 after 24 h of incubation at 37 °C.

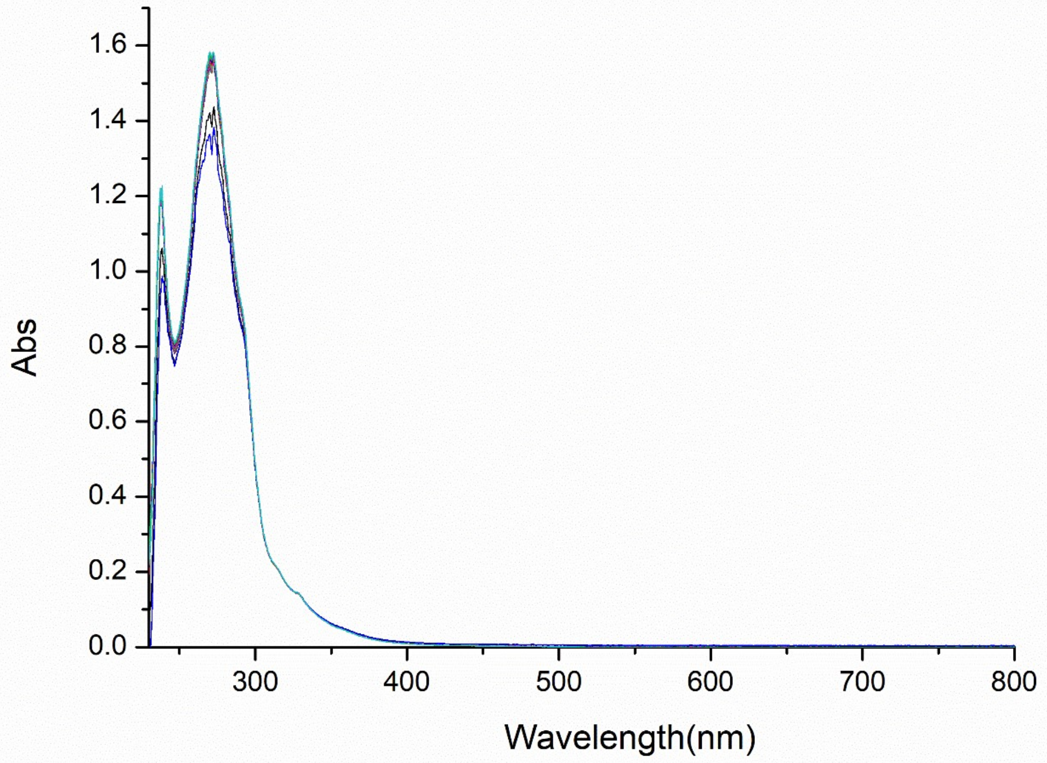

Supplementary Figure 7. UV-vis spectra of HSA with Au_2Phen (1:3 protein/gold ratio) in buffer phosphate.

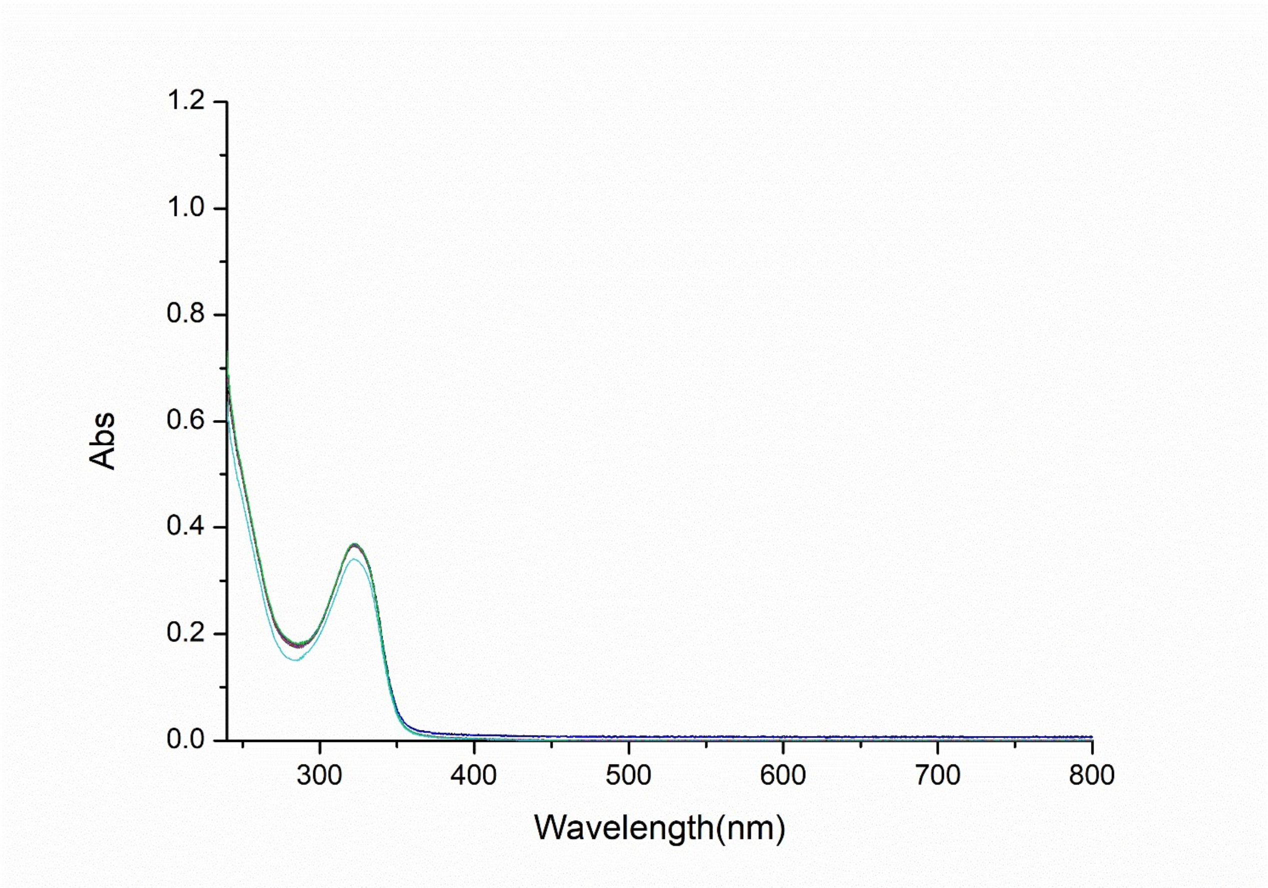

Supplementary Figure 8. UV-vis spectra of HSA with Aubipyc (1:3 protein/gold ratio) in buffer phosphate.

2 hCA I

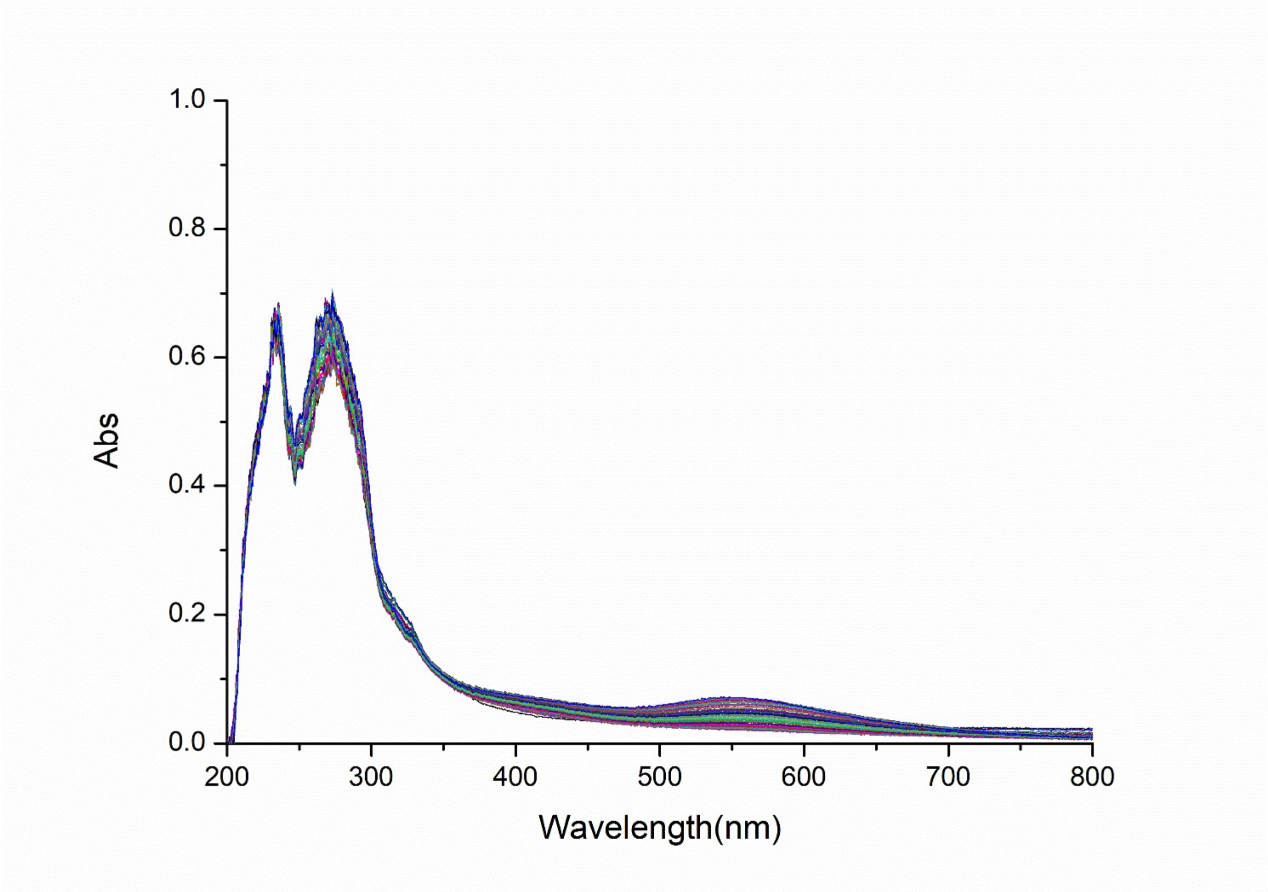

Supplementary Figure 9. UV-vis spectra of carbonic anhydrase with Au_2Phen (1:3 protein/gold ratio) in buffer phosphate.

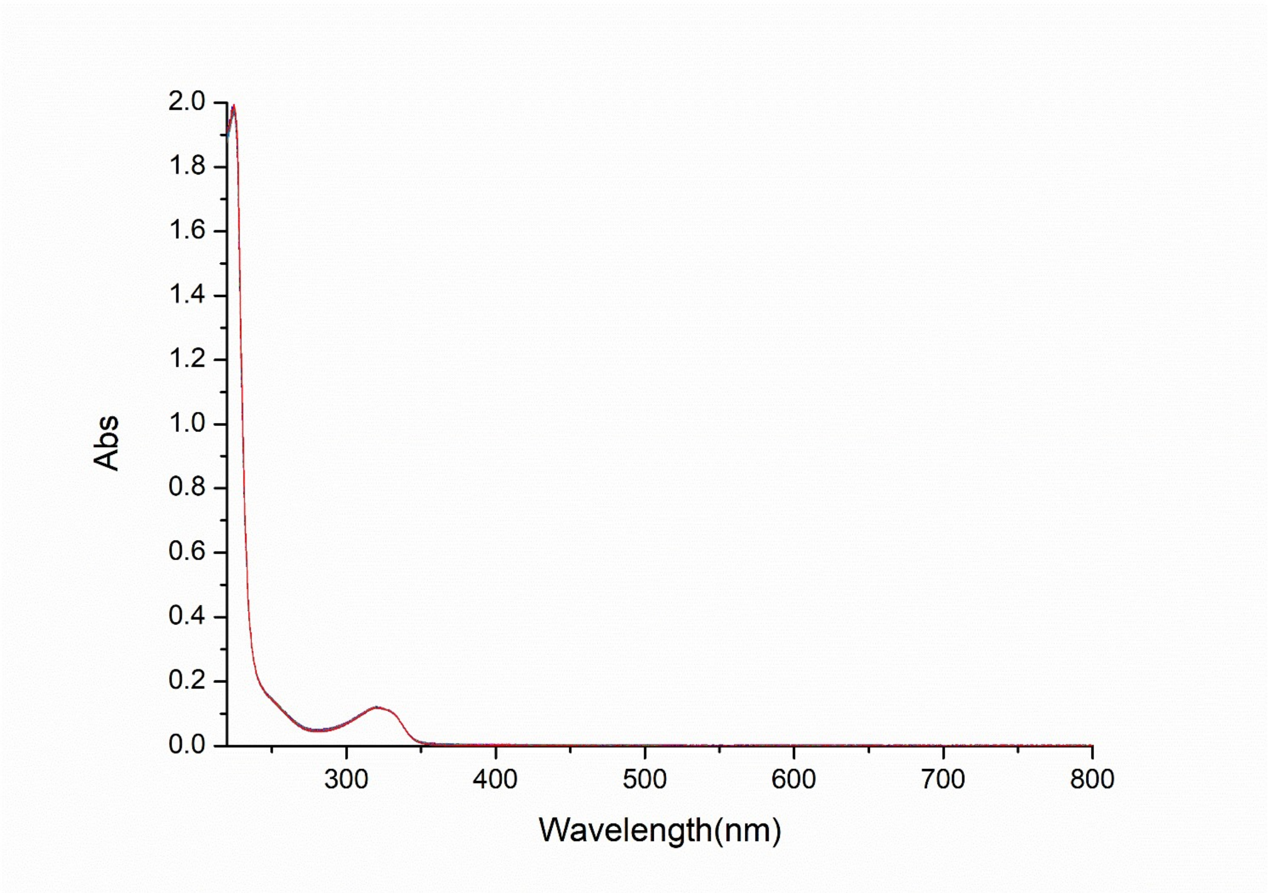

Supplementary Figure 10. UV-vis spectra of carbonic anhydrase with Aubipyc (1:3 protein/gold ratio) in buffer phosphate.

3 Peptide fragment of TrxR

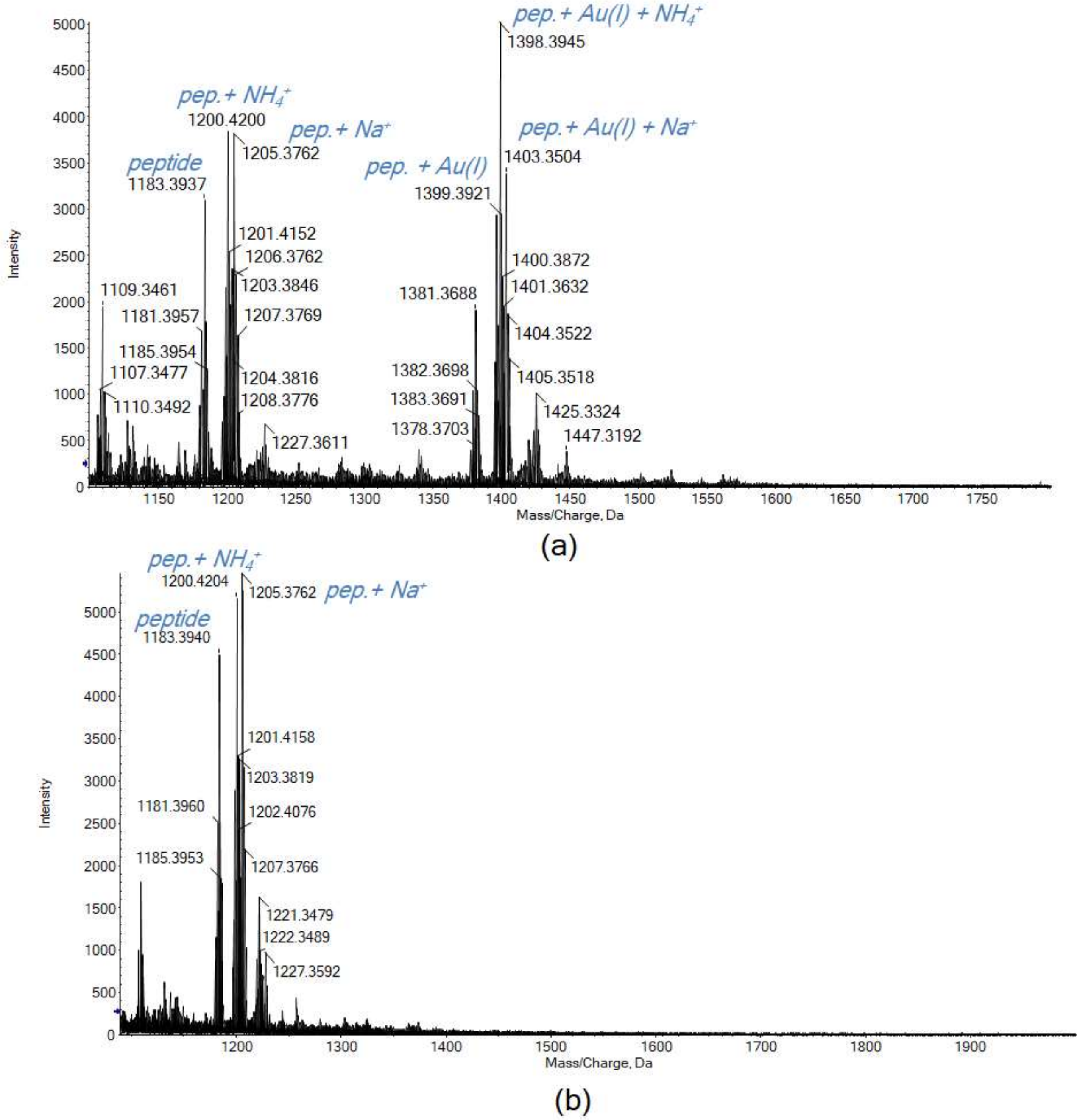

Supplementary Figure 11. Deconvoluted ESI Q TOF spectrum of dodecapeptide solution $5x10^{-7}$ M with DTT and Auoxo6 (1:10:1 peptide/reducing agent/gold ratio) in ammonium acetate solution 2 mM (pH 6.8) after a) 2 h and b) 24 h of incubation at 37 °C.

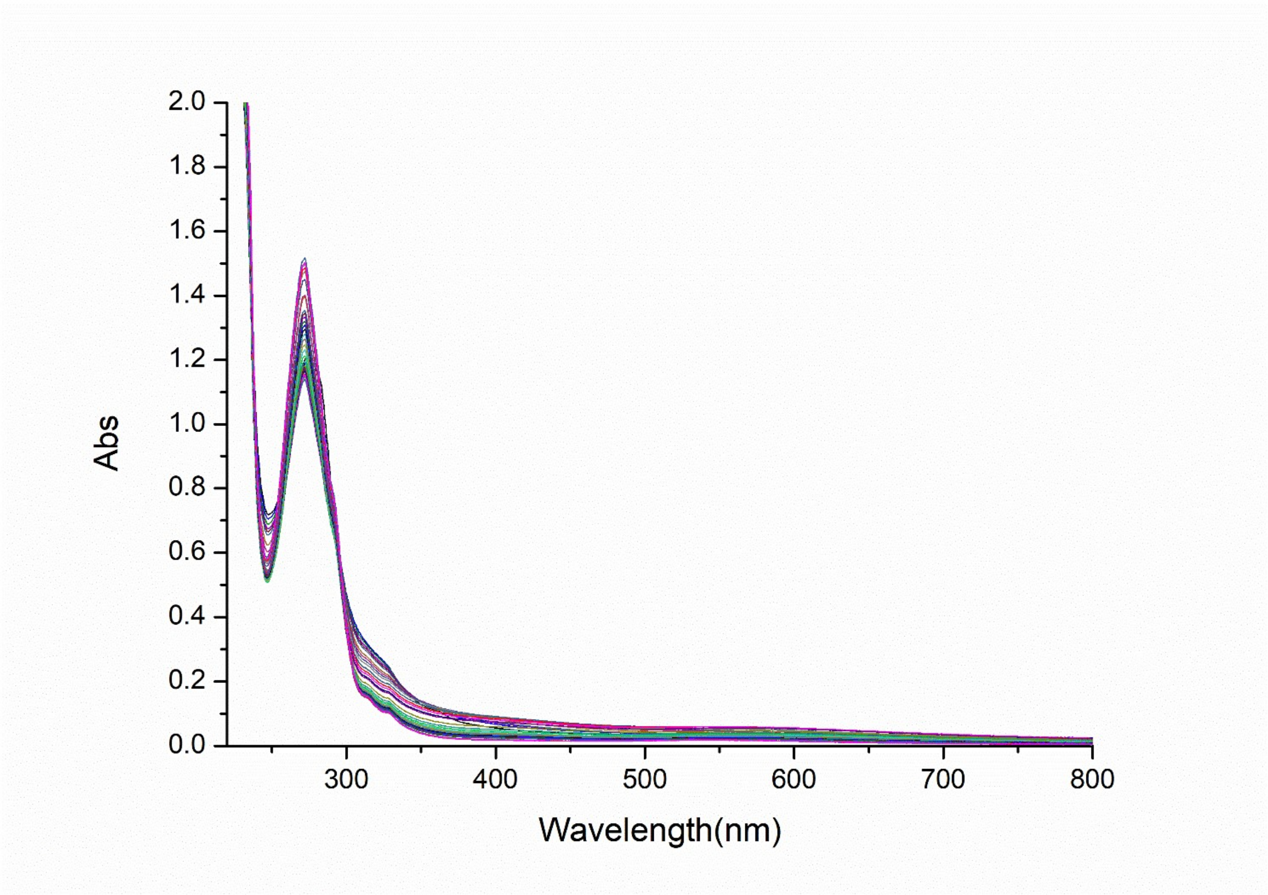

Supplementary Figure 12. UV-vis spectra of thioredoxin reductase dodecapeptide model with Au_2Phen (1:3 peptide/gold ratio) in buffer phosphate.

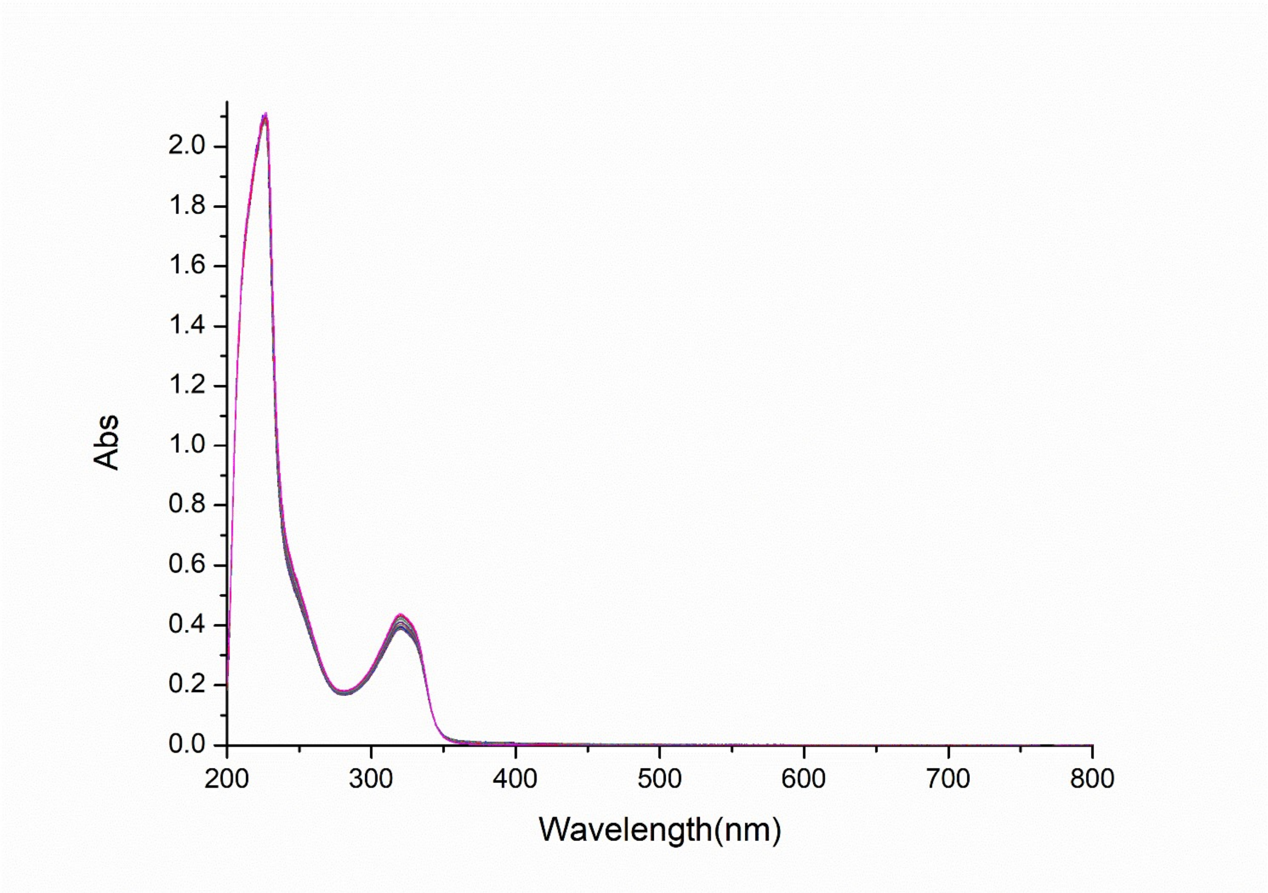

Supplementary Figure 13. UV-vis spectra of thioredoxin reductase dodecapeptide model with Aubipyc (1:3 peptide/gold ratio) in buffer phosphate.
